# Decreased reproducibility and abnormal experience-dependent plasticity of network dynamics in Fragile X circuits

**DOI:** 10.1038/s41598-020-71333-y

**Published:** 2020-09-03

**Authors:** Helen Motanis, Dean Buonomano

**Affiliations:** Departments of Neurobiology and Psychology, and Integrative Center for Learning and Memory, University of California, 630 Charles E Young Dr S, Center for Health Sciences Building, Los Angeles, CA 90095 USA

**Keywords:** Neuroscience, Diseases of the nervous system, Learning and memory, Neural circuits

## Abstract

Fragile X syndrome is a neurodevelopmental disorder associated with a broad range of neural phenotypes. Interpreting these findings has proven challenging because some phenotypes may reflect compensatory mechanisms or normal forms of plasticity differentially engaged by experiential differences. To help minimize compensatory and experiential influences, we used an ex vivo approach to study network dynamics and plasticity of cortical microcircuits. In *Fmr1*^*−/y*^ circuits, the spatiotemporal structure of Up-states was less reproducible, suggesting alterations in the plasticity mechanisms governing network activity. Chronic optical stimulation revealed normal homeostatic plasticity of Up-states, however, *Fmr1*^*−/y*^ circuits exhibited abnormal experience-dependent plasticity as they did not adapt to chronically presented temporal patterns in an interval-specific manner. These results, suggest that while homeostatic plasticity is normal, *Fmr1*^*−/y*^ circuits exhibit deficits in the ability to orchestrate multiple forms of synaptic plasticity and to adapt to sensory patterns in an experience-dependent manner—which is likely to contribute to learning deficits.

## Introduction

Fragile X syndrome (FXS) is the leading monogenic cause of autism and intellectual disabilities, and reflects loss of function mutations in the RNA binding protein, Fragile X Mental Retardation Protein (FMRP)^1–4^. Since the generation of the first mouse model of FXS^5^, a large number of neural phenotypes have been associated with the syndrome including: abnormalities in dendritic spine morphology and stabilization^6–12^, altered short- and long-term forms of synaptic plasticity^13–20^, abnormal axonal development^19,21,22^, changes in interneuronal connectivity^23,24^, channelopathies^16,25–31^, and imbalanced excitation/inhibition^24,32–44^.

The sheer diversity of reported neural phenotypes highlights the challenge in determining which phenotypes are a primary consequence of the absence of FMRP, from those that reflect indirect secondary neural phenotypes. These secondary neural phenotypes could reflect: (1) compensatory and homeostatic mechanisms, i.e., genetic redundancy and homeostatic plasticity engaged to compensate for the primary consequences of the lack of FMRP^44–46^; or (2) normal forms of plasticity shaped by experiential differences, including differences in sensory exploration, social interactions and maternal care. Indeed, it is increasingly recognized that some of the neural phenotypes reported in animal models of FXS and autism spectrum disorder (ASD) may reflect compensatory mechanisms or differential developmental experiences^44,45^. For example, it is well established that early differences in sensory experience, social interactions and maternal rearing, can alter numerous neural properties throughout life^47–53^. As a result of decreased social interactions, young FMRP-deficient animals could in effect inhabit an impoverished sensory environment. Indeed, it is also well established that in mouse models of FXS and ASD, animals experience differences in social interactions, sensory experience, and maternal care^54–59^. Thus, in addition to the possibility that some neural phenotypes may reflect compensatory plasticity, some phenotypes may be a consequence of normal forms of plasticity differentially shaped experiential differences.

One approach towards disentangling neural phenotypes that are a direct consequence of the absence of FMRP and those that reflect experiential differences is to study network activity and plasticity in isolated circuits that develop ex vivo. Cortical organotypic slices are ideally suited towards this goal since they maintain much of the neural circuitry features of in vivo cortical circuits and recapitulate many aspects of in vivo development^60–62^. For example, ex vivo development of organotypic slices recapitulates the transition from early quiescent networks, to networks that exhibit robust spontaneous activity and Up-states^63,64^. Furthermore, ex vivo cortical circuits have been shown to be capable of performing a number of interesting computations^65–69^. Thus, ex vivo circuits allow the study of network-level properties while ascertaining that any differential neural phenotypes emerge from within the circuit being studied, and decrease the likelihood that the observed phenotypes are a consequence of altered developmental experiences. Furthermore, compared to acute slices, ex vivo circuits are less susceptible to rearing and social interaction differences.

Among the best studied examples of network-level dynamics within cortical circuits are Up-states^70–73^, a term that generally refers to network-wide regimes in which neurons transition between quiescent Down-states and stable depolarized states with moderate firing rates that are actively maintained by recurrently generated positive feedback^74–76^. Up-states require the tuning of many cellular and synaptic properties including the ratios of excitation and inhibition^75,77–79^. Similar to in vivo circuits, Up-states in organotypic slices emerge over the course of ex vivo development^63,80,81^. Up-states have been proposed to have multiple functional roles, including memory consolidation and synaptic homeostasis^82–86^. And it has been hypothesized that Up-states correspond to the desynchronized regimes of awake cortex^87^.

A number of studies have examined Up-states in Fmr1-KO circuits as a means to dissect the network-level abnormalities involved in FXS^24,34,80,88–90^. Here, in order to help minimize experiential and compensatory effects, we examine the development and plasticity of Up-states in *Fmr1*^*−/y*^ circuits that developed ex vivo. Our results establish a sequence of developmental delays in *Fmr1*^*−/y*^ circuits, and importantly show that even when overall Up-state frequency is equivalent between WT and FX circuits, the spatiotemporal structure of the underlying activity is altered. These results are consistent with the notion that FXS may reflect the inability to properly orchestrate the multiple forms of plasticity required to generate stable and reproducible spatiotemporal patterns of neural activity^46^. We thus used all-optical technique to study how *Fmr1*^*−/y*^ circuits adapt to chronic stimulation meant to emulate sensory experience. We find that while homeostatic plasticity of Up-states is normal, a form of ex vivo experience-dependent plasticity, that emulates learning, is altered in Fmr1-KO circuits.

## Results

### *Fmr1*^*–/y*^ circuits exhibit a developmental delay in the emergence of spontaneous activity

**Figure 1:**
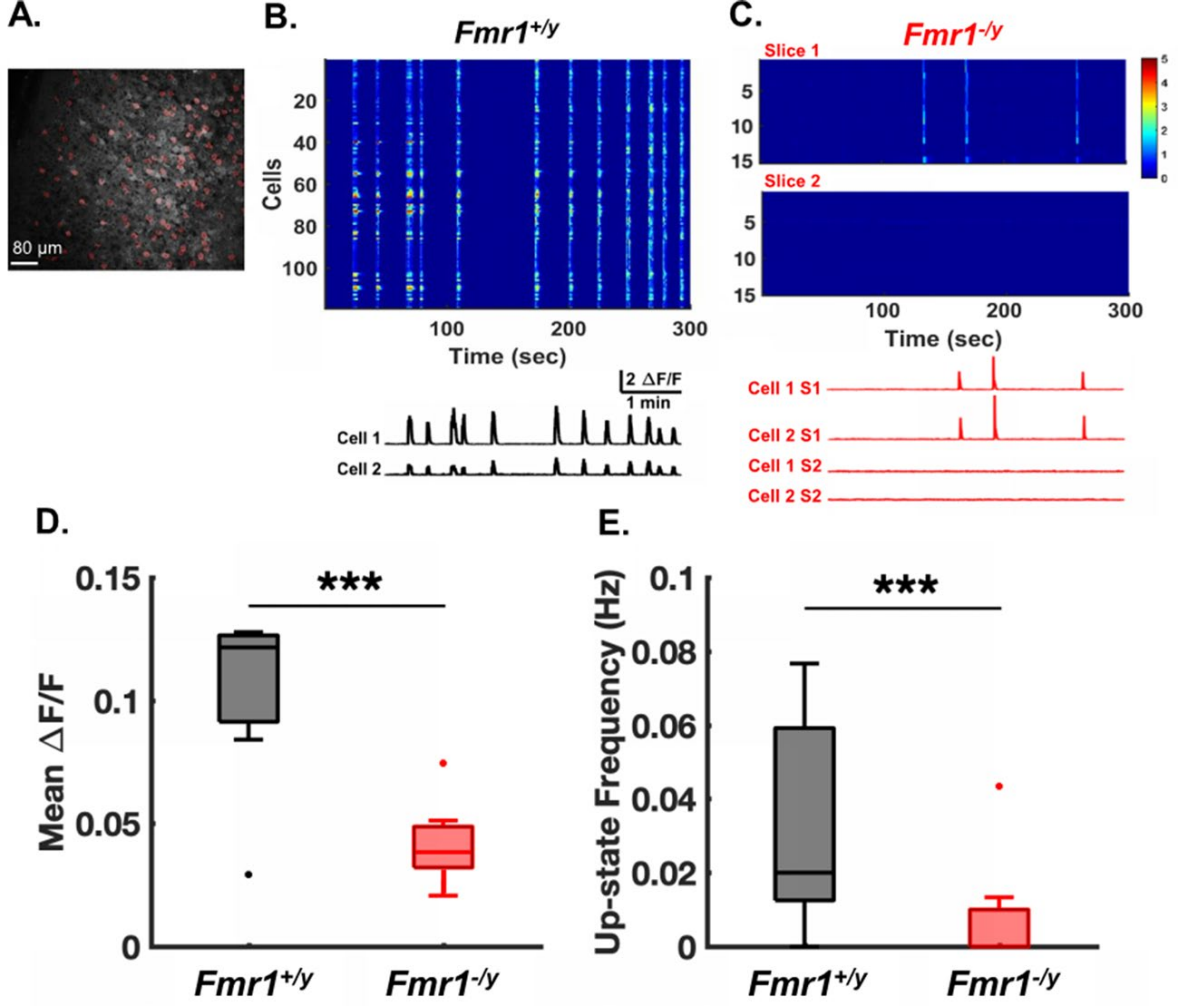
* Fmr1*^*−/y*^ circuits exhibit reduced Up-state frequency at 11–16 DIV. (**A**) Representative field of view of two-photon Ca^2+^-imaging experiment in an ex vivo slice of the auditory cortex. Red filled circles represent regions of interest (ROIs) of selected neurons. (**B**) Top: Sample ΔF/F signals of 119 neurons from a *Fmr1*^+*/y*^ slice. Note that spontaneous Up-states appear as bouts of synchronous activity across the network. Bottom: Two example traces of changes in GCaMP6f fluorescence intensity (∆F/F) from the *Fmr1*^+*/y*^ slice. (**C**) Same as in B for two separate *Fmr1*^*−/y*^ slices. The activity of the first 15 neurons is shown. (**D**) Mean ΔF/F was significantly reduced in *Fmr1*^*−/y*^ circuits (0.04 ± 0.004, n = 10) compared to *Fmr1*^+*/y*^ circuits (0.11 ± 0.019, n = 9; Mann–Whitney test, p = 0.001). (**E**) Up-state frequency is significantly lower in *Fmr1*^*−/y*^ (0.006 ± 0.004 Hz n = 10) compared to *Fmr1*^+*/y*^ ex vivo circuits (0.034 ± 0.009 Hz, n = 9; Mann–Whitney test, p = 0.004). Here, and for all subsequent box-and-whisker plots, the box boundaries represent the 25–75% (interquartile) range, whiskers represent the range of the non-outlier data points. Outliers (squares) are those that fall 1.5 times the interquartile range above or below the box edges. Lines within the box represents the median.

**Figure 2:**
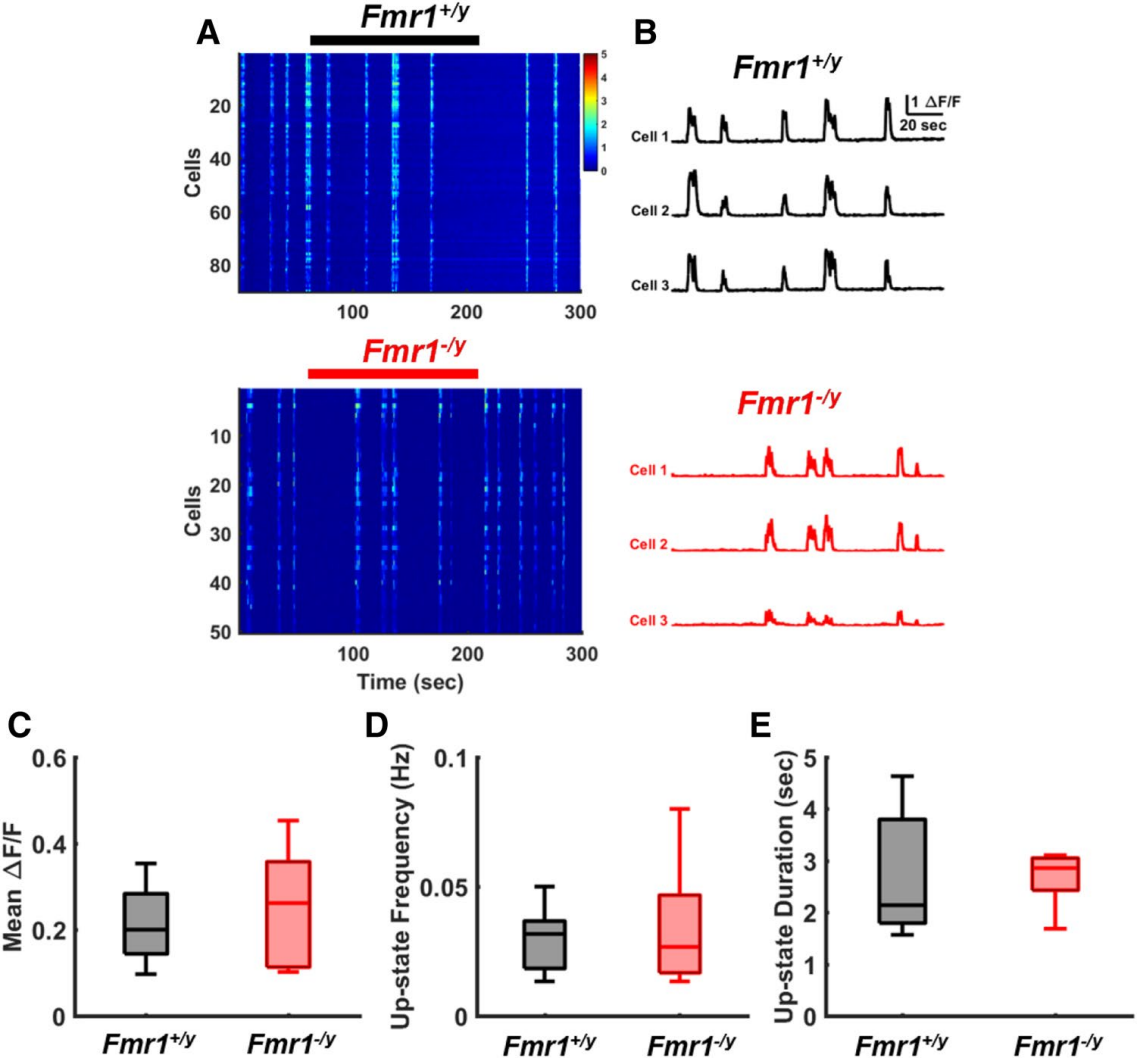
Normal network activity and Up-state frequency in *Fmr1*^*−/y*^ circuits at 25–30 DIV. (**A**) Five-minute heat map of ∆F/F signals from a *Fmr1*^+*/y*^ ex vivo circuit (top: n = 90) and from a *Fmr1*^*−/y*^ circuit (bottom: n = 50). (**B**) Example ∆F/F traces for 6 representative neurons at 25–30 DIV. The upper and bottom 3 neurons are from a *Fmr1*^+*/y*^ and *Fmr1*^*−/y*^ slice, respectively. Bold lines above heat maps in (**A**) (black for WT and red for KO) correspond to the traces in (**B**). (**C**) Mean ΔF/F of *Fmr1*^+*/y*^ circuits (0.21 ± 0.03, n = 8) was not different from *Fmr1*^*−/y*^ circuits (0.24 ± 0.04, n = 11; t-test, p = 0.52). (**D**) Frequency of Up-states was not different between *Fmr1*^+*/y*^ (0.02 ± 0.004 Hz) and *Fmr1*^*−/y*^ ex vivo slices (0.03 ± 0.006 Hz; t-test, p = 0.55). (**E**) Up-states duration was not different between *Fmr1*^+*/y*^ (2.70 ± 0.42 s) and *Fmr1*^*−/y*^ ex vivo slices (2.94 ± 0.35 s; Mann–Whitney test, p = 0.54).

Studies in acute slices and in vivo have reported both cortical hyper- and hypoactivity in Fmr1-KO animals^4,24,34,38,89,91,92^. We thus first examined the emergence of spontaneous network activity in auditory cortical slices that developed ex vivo. We contrasted *Fmr1*^*−/y*^ and *Fmr1*^+*/y*^ circuits using two-photon Ca^2+^-imaging at two ex vivo developmental ages: 11–16 DIV and 25–30 DIV (Figs. 1, 2). As exemplified in Fig. 1A,B, GCaMP6f fluorescence across 119 neurons from a *Fmr1*^+*/y*^ circuit revealed spontaneous network-wide events. We refer to these network-wide events as Up-states, and have confirmed with whole-cell electrophysiology that these events reflect discrete shifts from a quiescent state in which membrane voltage is close to resting, to depolarized regimes with low firing rates (e.g., Fig. Supplement 1)^64,80^. *Fmr1*^*−/y*^ circuits at 11–16 DIV exhibited reduced activity (Fig. 1C) and mean ΔF/F (0.04 ± 0.004, n = 10) compared to *Fmr1*^+/y^ circuits (0.11 ± 0.019, n = 9; Mann–Whitney test, p = 0.001; Fig. 1D). Up-state frequency was also significantly decreased in *Fmr1*^*−/y*^ (0.006 ± 0.004 Hz, n = 10) compared to *Fmr1*^+*/y*^ circuits (0.034 ± 0.009 Hz, n = 9; Mann–Whitney test, p = 0.004; Fig. 1E). Because *Fmr1*^*−/y*^ circuits did not exhibit much activity and because Up-state frequency was very low, the Up-state duration was only calculated for *Fmr1*^+*/y*^ circuits (3.25 + 0.58 s, n = 9; data not shown).

Similar experiments in older slices, revealed that by 25–30 DIV the deficit in network activity had normalized (Fig. 2A,B). That is, there was no difference in mean ΔF/F between *Fmr1*^+*/y*^ (0.21 ± 0.03, n = 8) and *Fmr1*^*−/y*^ circuits (0.24 ± 0.04, n = 11; Fig. 2C). Similarly, Up-state frequency and duration were not significantly different between WT (0.02 ± 0.004 Hz, 2.70 ± 0.42 s, n = 8) and *Fmr1*^*−/y*^ circuits (0.03 ± 0.006 Hz, 2.94 ± 0.35 s, n = 11; Fig. 2D,E). As evident from the ΔF/F traces (Fig. 2A,B), and as expected during Up-states, Ca^2+^ signals were tightly correlated, but there was no significant difference in the mean cell pairwise correlations between *Fmr1*^*−/y*^ (Fisher-transformed 0.63 ± 0.03, n = 11) and *Fmr1*^+*/y*^ circuits at 25–30 DIV (Fisher-transformed 0.70 ± 0.05, n = 8; data not shown).

### Spatiotemporal pattern of activity in *Fmr1*^*−/y*^ circuits is less reproducible

**Figure 3:**
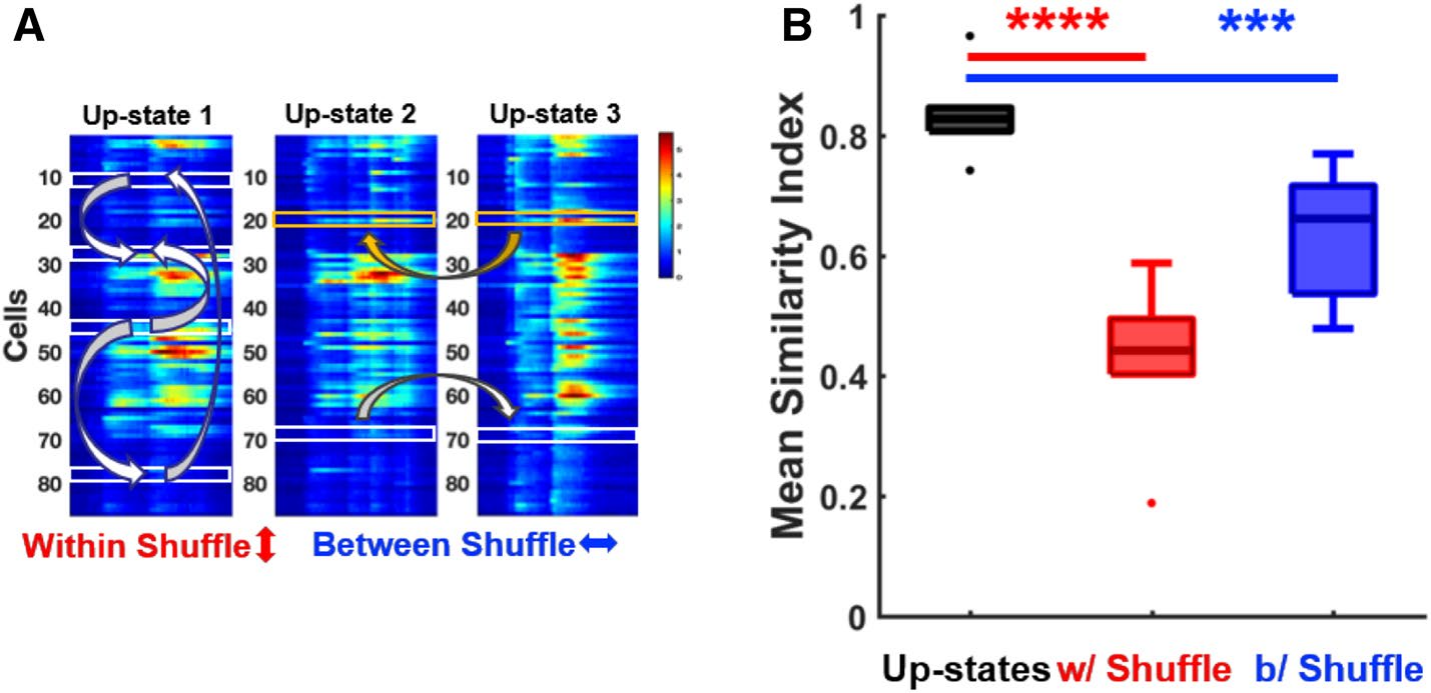
Up-states in WT circuits exhibit spatiotemporal structure. (**A**) Three Up-states from a WT circuit. The ‘Within shuffle’ procedure consists of shuffling cells (marked with white boxes) within each Up-state. The ‘Between shuffle’ procedure consists of shuffling between Up-states while maintaining cell identity. The similarity index is then calculated by determining the mean pairwise correlation between Up-states (see “Methods”). (**B**) A one-way ANOVA across the mean similarity index of the three groups revealed a significant effect (F_2,15_ = 23.85, p < 10^–4^). Group WT mean similarity index of Up-states (0.83 ± 0.03; n = 6, black, most to the left group) is significantly higher than the mean similarity index of within shuffled Up-states (0.41 ± 0.05; t-test, p < 0.0001, red, middle group) and between shuffled Up-states (0.62 ± 0.03; t-test, p = 0.001, blue, most to the right group).

These results demonstrate that ex vivo circuits exhibited a signature developmental delay of FXS and suggested that by the 4th week of ex vivo development Up-state dynamics were normal. The term Up-states is used to refer to a number of interrelated forms of neural dynamics, and is sometimes interpreted to refer to bi-stable shifts from a quiescent to active state^70,93^. However, it has been shown that spontaneous and evoked Up-states, in vivo*,* in vitro, and ex vivo, can exhibit reproducible spatiotemporal structure^63,72,73,77,94^. To determine if this was the case ex vivo, we first determined whether there were spatiotemporal patterns of activity within Up-states in WT circuits at 25–30 DIV. In other words, are there distinct spatiotemporal patterns of activity embedded within Up-states that are repeatedly replayed across Up-states? Such spatiotemporal structure could take various forms, including that some neurons could consistently tend to fire at the start of an Up-state while others at the end. To determine if there was detectable structure to the Up-states, we extracted each spontaneous Up-state during an experiment (Fig. Supplement 2A), and calculated the mean similarity (Fig. 3, see “Methods”) across all Up-state pairs in an experiment. We also estimated the similarity index expected by chance based on the statistics of the empirically observed Up-states. Towards this end we shuffled cells within the same Up-state (w/Shuffle), as well between Up-states (b/Shuffle) (Fig. 3, see “Methods”). A one-way ANOVA across the unshuffled and two shuffled conditions revealed that in WT slices there was a significant difference in mean similarity (F_2,15_ = 23.85, p < 10^–4^), and that the unshuffled mean similarity index (0.83 ± 0.03, n = 6) was significantly higher than in the shuffled controls (w/ Shuffle, 0.41 ± 0.05; t-tests on Fisher transformed data, p < 10^–4^ and b/Shuffle, 0.62 ± 0.03; p = 0.001; Fig. 3B). Visual inspection and clustering further confirms this notion and reveal that Up-states exhibit distinct structure (Fig. Supplement 2B,C). These results establish that Up-states in WT ex vivo circuits at 25–30 DIV exhibit reproducible spatiotemporal structure.

**Figure 4:**
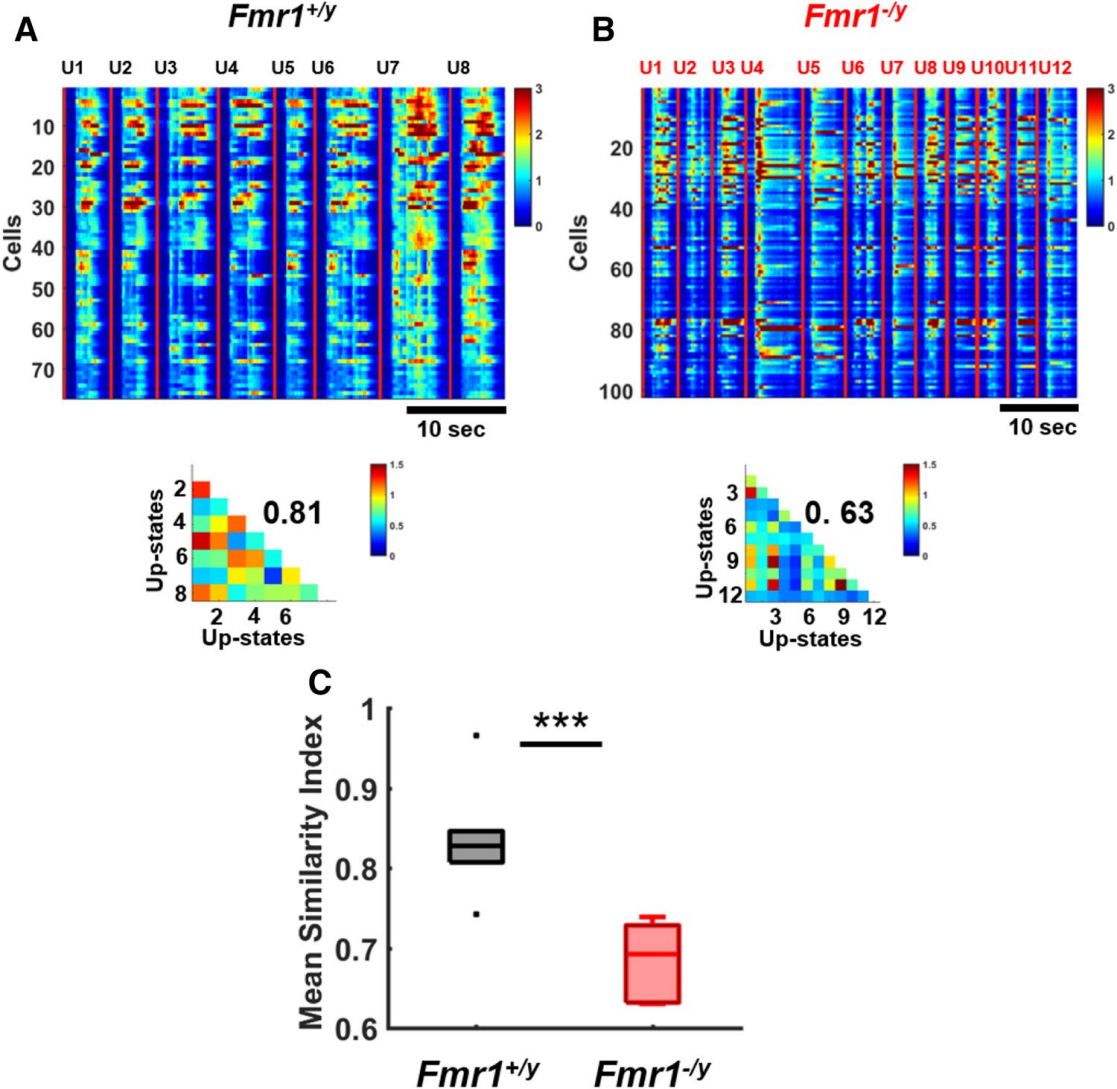
Spatiotemporal patterns of Up-states of *Fmr1*^*−/y*^ circuits are more variable. (**A**) Heat maps of eight concatenated Up-states from a *Fmr1*^+*/y*^ circuit. Triangles below heat maps represent the pairwise similarity index between all Up-states, and the overall mean of similarity indices is in black (0.81). (**B**) Twelve concatenated Up-states from a *Fmr1*^*−/y*^ circuit, and similarity indices (mean of 0.63). (**C**) Group mean similarity index of Up-states in *Fmr1*^+*/y*^ circuits (0.83 ± 0.03; n = 6) was significantly higher than mean similarity index of Up-states in *Fmr1*^*−/y*^ circuits (0.68 ± 0.01; n = 7; Mann–Whitney test, p = 0.001).

Having established the presence of spatiotemporal structure in WT circuits, we next asked whether the structure and reproducibility of Up-states in *Fmr1*^*−/y*^ circuits was similar to WT circuits. As above, we calculated the pairwise similarity of all Up-states within each slice (Fig. 4A,B). Group analysis revealed that *Fmr1*^*−/y*^ circuits exhibited a significantly reduced mean similarity index (0.68 ± 0.01, n = 7) compared to *Fmr1*^+*/y*^ circuits (0.83 ± 0.03, n = 6; Mann–Whitney, p = 0.001; Fig. 4C), indicating that the variability of Up-states in *Fmr1*^*−/y*^ ex vivo circuits was higher—in other words, the spatiotemporal patterns of activity in FX KO circuits were less stable and less reproducible. We further confirmed that this finding is robust to parameter assumptions or the ΔF/F measure. For example, similarity indices obtained on the z-score fluorescence also revealed that Up-state reproducibility in WT circuits (0.70 ± 0.02, n = 7) was significantly higher than in *Fmr1*^*−/y*^ circuits (0.54 ± 0.03, n = 6; t-test, p = 0.001, data not shown). A question that arises from these results is whether Up-states reproducibility undergoes developmental changes. We thus compared similarity index of Up-states from circuits recorded at 11–16 DIV and 25–30 (this analyses cannot be performed in the *Fmr1*^*−/y*^ circuits because of the lack of Up-states at 11–16 DIV). Mean similarity index of young (11–16 DIV) Up-states of WT circuits was not significantly different (0.72 ± 0.11, n = 8) from mean similarity index of Up-states of WT circuits at 25–30 DIV (0.83 ± 0.03, n = 6; t-test, p = 0.4), and thus, the structure present in older slices was also present in the younger circuits. Overall, these findings demonstrate that even though the levels of spontaneous activity normalized by the 4th week of ex vivo development, the stability and reproducibility of spontaneous neural dynamics in FMRP-deficient circuits was compromised. In other words, while average levels of activity have normalized the structure of this activity is different in *Fmr1*^*−/y*^ circuits.

### Chronic optical stimulation induces normal homeostatic plasticity of up-states

**Figure 5:**
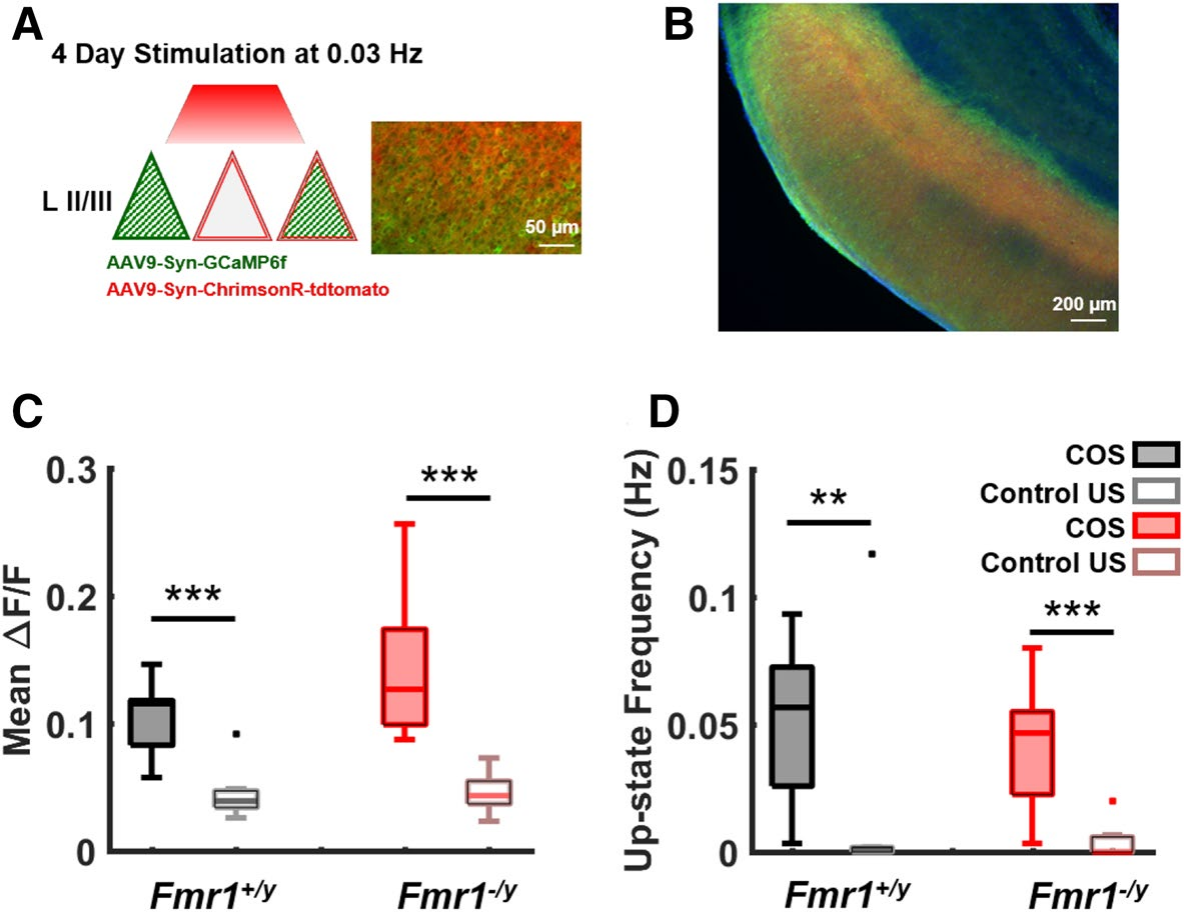
Normal homeostatic plasticity of spontaneous activity in *Fmr1*^*−/y*^ circuits. (**A**) Schematic of the all-optical chronic stimulation paradigm used for the induction of homeostatic plasticity of spontaneous activity. Slices co-expressing GCaMP6f and ChrimsonR were stimulated for 4 days. Left panel shows co-expression of ChrimsonR-tdTom (double lines in red) expression with GCaMP6f (green stripes inside triangles) somatic expression. (**B**) DAPI stained slice showing ChrimsonR-tdTom (red) expression with GCaMP6f (green) somatic expression. (**C**) Spontaneous activity was measured from COS (chronic optical stimulation) and control (unstimulated) slices using two-photon Ca^2+^-imaging. Mean ∆F/F was significantly reduced following COS in both *Fmr1*^+*/y*^ and *Fmr1*^*−/y*^ circuits (Two-way ANOVA: F_1,24_ = 33.16, p = 10^–6^), with no significant interaction between genotype and stimulation. A significant reduction in mean ∆F/F of COS *Fmr1*^+*/y*^ circuits (0.04 ± 0.008, n = 7) was found compared to control *Fmr1*^+*/y*^ (0.1 ± 0.01, n = 7; t-test, p < 0.001). A similar reduction was also found in the mean ∆F/F of COS *Fmr1*^*−/y*^ (0.04 ± 0.006, n = 7) compared to control *Fmr1*^*−/y*^ (0.14 ± 0.02, n = 7; t-test, p = 0.001). Black filled boxes (most to the left) are from control *Fmr1*^+*/y*^ slices, gray empty boxes (second to the left) are from COS *Fmr1*^+*/y*^, red filled boxes (third to the left) are from control *Fmr1*^*−/y*^ and pink empty boxes (most to the right) are from COS *Fmr1*^*−/y*^. (**D**) Similarly, COS resulted in a significant reduction of Up-state frequency in both genotypes: Up-state frequency of COS *Fmr1*^+*/y*^ (0.01 ± 0.01, n = 7) was significantly reduced compared to control *Fmr1*^+*/y*^ (0.05 ± 0.01, n = 7; Mann–Whitney, p = 0.01). A similar reduction in Up-state frequency was found in COS *Fmr1*^*−/y*^ (0.004 ± 0.002, n = 7) compared to control *Fmr1*^*−/y*^ (0.04 ± 0.009, n = 7; Mann–Whitney test , p = 0.004).

The fact that Up-states are observed both in vivo, in acute slices, and ex vivo, strongly suggests that they represent a fundamental dynamic regime that cortical microcircuits are ontogenetically programmed to seek out. Consistent with this notion, Up-states are homeostatically regulated^64,80,95^. Thus the developmental delay and decreased reproducibility of Up-states observed in *Fmr1*^*−/y*^ circuits could reflect abnormal forms of homeostatic plasticity—or more generally deficits in the ability of cortical microcircuits to adapt in a flexible manner to natural fluctuations and external input. To address this issue we used an all-optical method to chronically stimulate and interrogate cortical circuits to study homeostatic regulation of network dynamics. Slices co-expressing the red-shifted opsin, ChrimsonR, and GCaMP6f. were optically stimulated for 3–4 days at 21–25 DIV (Fig. 5A,B). A concern, however, with this all-optical method is the potential interaction between GCaMP6f and ChrimsonR activation—particularly whether ChrimsonR-positive cells are activated during two-photon scanning. To directly examine this issue, we performed simultaneous whole-cell recording and two-photon Ca^2+^-imaging of cells co-expressing GCaMP6f and ChrimsonR (Fig. Supplement 3). In 4/4 independent experiments, onset of two-photon scanning (920 nm) did not activate ChrimsonR-positive cells as can be seen by the absence of a voltage deflection of the membrane potential at laser onset (Bottom black trace; Fig. Supplement 3).

Chronic optical stimulation (COS) consisting of a single 50 ms pulse of light every 30 s for 3–4 days produced powerful homeostatic plasticity of Up-states in both *Fmr1*^*−/y*^ and *Fmr1*^+*/y*^ ex vivo circuits at 25–30 DIV. Specifically, comparison of stimulated and unstimulated control circuits revealed a strong decrease in the mean ∆F/F in both genotypes (Fig. 5C). Importantly, COS also resulted in a significant reduction of Up-state frequency in both genotypes (Fig. 5D). Since chronically optically stimulated slices exhibited very few Up-states, event duration was calculated only for the unstimulated control groups and similar to results in Fig. 2E there was no significant difference between Up-state duration of unstimulated control *Fmr1*^+*/y*^ circuits (5.41 ± 1.25 s, n = 7) and *Fmr1*^*−/y*^ circuits (8.08 ± 2.47 s, n = 7; data not shown). These results demonstrated that: (1) low frequency (0.033 Hz) chronic optical stimulation produced a profound decrease in Up-state frequency, thus confirming that Up-states are powerfully regulated by external activity; and (2) that homeostatic plasticity of overall network level Up-states appears to be normal in *Fmr1*^*−/y*^ circuits.

### ***Fmr1***^***−/y***^ circuits exhibit an impaired ability to adapt to the temporal structure of chronically-presented external stimuli

**Figure 6:**
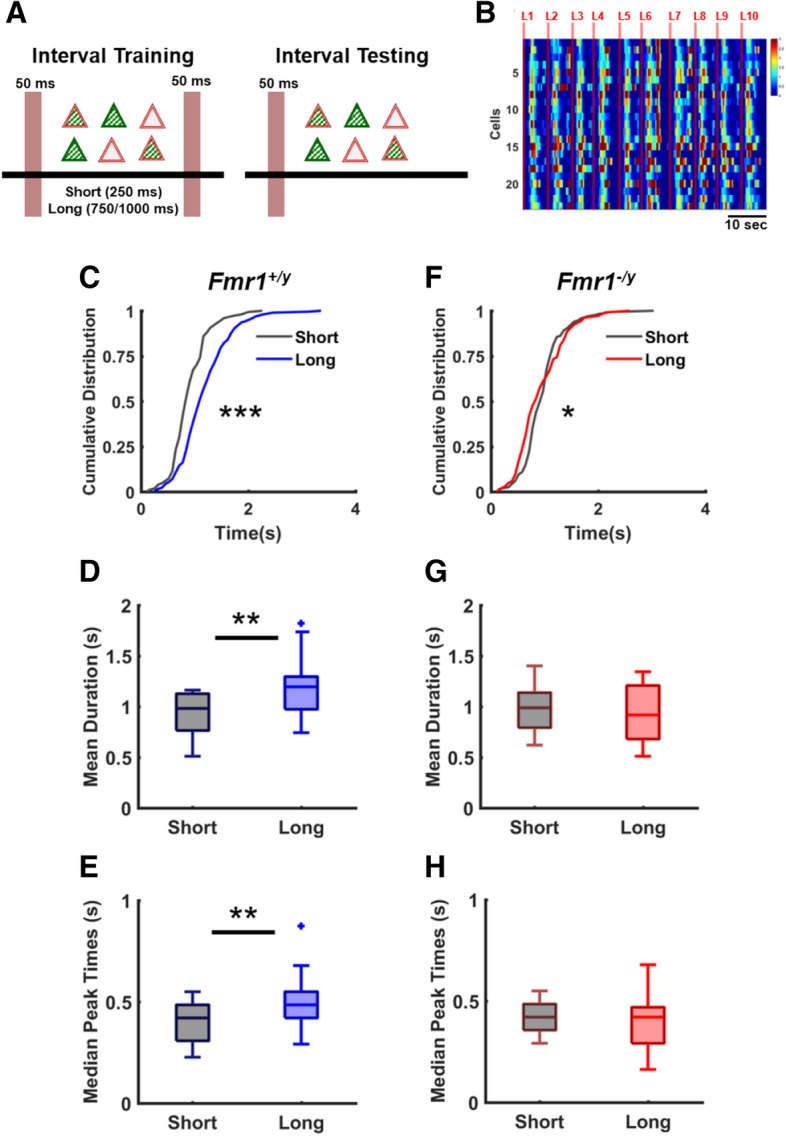
Interval learning is abnormal in *Fmr1*^*−/y*^ circuits. (**A**) Schematic of the interval learning training and testing paradigm. Slices co-expressing GCaMP6f (green stripes inside triangles) and ChrimsonR (double line in red, surface of triangles) were stimulated with two light pulses separated by short or long interval and then tested using a single pulse. (**B**) Heat maps of ten concatenated light-evoked responses during testing phase from a *Fmr1*^+*/y*^ circuit that was trained with 250 ms interval (short). (**C**) Cumulative distribution of duration of evoked light responses for *Fmr1*^+*/y*^ circuits was significantly different between circuits trained with short compared to long intervals (250: n = 150, 1,000: n = 187, K-S test, p < 10^–6^). (**D**) Mean duration of evoked responses of WT circuits trained with short intervals was significantly reduced (0.91 ± 0.05, n = 15) compared to long-trained WT circuits (1.18 ± 0.06, n = 19; t-test, p < 0.004). (**E**) Average median peak times of WT circuits trained with short intervals was significantly reduced (0.39 ± 0.02, n = 15) compared to circuits trained with long intervals (0.49 ± 0.03, p = 0.01, n = 19; t-test, p = 0.01). (**F**) Same as C but for *Fm1*^*−/y*^ circuits. Distributions were significantly different but did not maintain the expected differential effect between short and long trained at all points (250: n = 166, 1,000: n = 105, K-S test, p = 0.01). (**G**) Mean duration of evoked responses trained on short intervals (0.97 ± 0.05, n = 17) was not different than long intervals (0.93 ± 0.08, n = 11; t-test, p = 0.62) in *Fmr1*^*−/y*^ circuits. (**H**) Median peak times of *Fmr1*^*−/y*^ circuits trained with short intervals was not different (0.41 ± 0.01, n = 17) than the ones trained with long intervals (0.39 ± 0.04, n = 11; t-test, p = 0.61).

As a learning disability, FXS is defined in part by deficits in the ability to learn^38,96^, in other words, in the ability of neural circuits to properly adapt in response to the spatiotemporal structure of the patterns they experience. As *Fmr1*^*−/y*^ circuits appeared to adapt normally to unstructured external stimulation, we next asked whether *Fmr1*^*−/y*^ cortical circuits exhibit deficits in their ability to adapt to structured stimuli. In essence, whether these circuits undergo what can be considered a form of ex vivo learning^69^. As above we used an all-optical approach to stimulate and interrogate circuits. Ex vivo circuits expressing ChrimsonR and GCaMP6f were trained on an interval paradigm that consisted of two light pulses separated by short or long intervals every 60 s for a duration of 24 h (Fig. 6A). Immediately after training, ten pulses of a single red light were delivered to test evoked network dynamics (Fig. 6B). *Fmr1*^+*/y*^ circuits exhibited what can be considered a form of experience-dependent learning: the spatiotemporal structure of the evoked dynamics was dependent on the temporal structure of the experienced stimuli (Fig. 6B–E). Across all WT slices the distribution of evoked Up-states was significantly shorter in the group trained with short intervals compared to the ones trained with long intervals (shifted to the right) (Kolmogorov–Smirnov test, p < 10^–6^, Fig. 6C). Importantly, group data revealed that in *Fmr1*^+*/y*^ circuits the mean Up-states duration were significantly reduced in slices trained with short intervals (0.91 ± 0.05 s, n = 15) compared to slices trained on the long intervals (1.18 ± 0.06 s, n = 19; t-test, p < 0.004; Fig. 6D). Note that while there is an interval specific effect in WT circuits, it is not the case that the duration of the evoked Up-states closely match the trained duration—this is in part expected because of the nonlinear and slow Ca^2+^ dynamics filters the actual spike patterns and because slices are trained in the incubator in culture media and tested on the rig in ACSF. To further examine the effects of interval training on the spatiotemporal structure of the evoked dynamics, we extracted the peak times (the latencies, see “Methods”) for each cell across all evoked Up-states and contrasted the median peak times in *Fmr1*^+*/y*^ slices trained on the short and long intervals. This analysis also revealed a significant interval effect (short: 0.39 ± 0.02 s, n = 15, versus long: 0.49 ± 0.03 s, n = 19; t-test, p = 0.01; Fig. 6E).

In contrast to WT circuits that showed differential experience-dependent responses, in the *Fmr1*^*−/y*^ circuits there was no significant difference in the mean Up-state duration between the groups trained with short and long intervals (0.97 ± 0.05 s, n = 17 versus 0.93 ± 0.08, n = 11; t-test, p = 0.62; Fig. 6G)—although there was a small difference in the shape of the distribution but as seen in Fig. 6F there was no clear differential effect of the interval training protocol that was used, meaning that the distribution of the long group was not always longer (shifted to the right) (Kolmogorov–Smirnov test, p = 0.01). Additionally, there was no difference in the median peak times between short and long trained groups (0.41 ± 0.01, n = 17 versus 0.39 ± 0.04, n = 11; t-test, p = 0.61; Fig. 6H). We also contrasted these results using a two-way ANOVA, which revealed a significant interaction between the genotype and interval effects for mean Up-state duration and median peak times (F_1,58_ = 5.94, p = 0.01, F_1,58_ = 4.32, p < 0.05; respectively) and the absence of main effects. Thus, further confirming that *Fmr1*^*−/y*^ circuits did not differentially adapt to the different intervals. Together these results are the first to show that while ex vivo *Fmr1*^*−/y*^ circuits exhibit normal ability to homeostatically adapt to changes in external input, they lack the ability to adapt appropriately to the spatiotemporal structure of experienced stimuli—i.e., that FMRP-deficient ex vivo circuits exhibit a deficit in their ability to adapt to experienced stimuli. However, we stress that we cannot explicitly state that the WT circuits are specifically learning the time of the trained intervals, and that *Fmr1*^*−/y*^ circuits are not. Rather, we can state that the different training intervals differentially shape the dynamics of WT circuits, and that no such effects were observed in *Fmr1*^*−/y*^ circuits.

## Discussion

Ultimately the cognitive and behavioral deficits that characterize FXS are expressed at the level of abnormal neural network function and altered patterns of neural activity. While it is known that at the molecular level FXS is caused by the absence of FMRP, to date, the key neurophysiological deficits that ultimately underlie FXS remain unknown^4,97^. The cause of network-level alterations may eventually be traced to a single synaptic or cellular phenotype, or perhaps more likely, to the interaction between many different neural phenotypes. To date, however, the sheer diversity of neural phenotypes associated with FXS has led to the recognition that some neural phenotypes may reflect indirect experiential or compensatory mechanisms^44,45^. Here, our focus on plasticity of ex vivo network dynamics offers an approach to study the net effect of many different affected loci on network function. This ex vivo approach offers some novel advantages, such as decreasing the potential influence of some compensatory mechanisms and of potential experiential differences. However, it remains the case that even ex vivo neural phenotypes could arise as a result of secondary compensatory mechanisms engaged in response to the primary consequences of the lack of FMRP. Temporally constrained manipulations using CRISPR or siRNA will offer valuable tools to help further address this possibility in future studies.

Consistent with the neurodevelopmental delays observed in FXS patients and animal models of the disease^9,21,32,36,98–102^, we observed a delay in the emergence of Up-states in ex vivo *Fmr1*^*−/y*^ circuits. The presence of a delay during ex vivo developmental further supports the notion that the lack of FMRP does indeed alter the temporal profile of the ontogenetic program underlying neurodevelopment. However, the delayed development of Up-states that we observed was not a simple developmental delay that eventually completely normalized to match WT dynamics. Even after Up-state frequency and duration normalized, there was increased variability of the spatiotemporal structure of spontaneous network dynamics (Fig. 4). It is difficult to infer the consequences of such increased variability, but this phenotype could be interpreted as impairment in the ability of specific patterns to “consolidate”, and occur as a result of the inability of multiple different forms of plasticity to interact in an orchestrated manner.

As a first step towards testing the hypothesis that specific forms of network-level plasticity may be impaired in *Fmr1*^*−/y*^ circuits, we examined the homeostatic regulation of Up-state frequency during late ex vivo development. Chronic optical stimulation over a period of days revealed a robust decrease in spontaneous network dynamics in both WT and *Fmr1*^*−/y*^ circuits. These results indicate that mature *Fmr1*^*−/y*^ circuits seem to exhibit normal homeostatic plasticity of spontaneous Up-states, suggesting that the mechanisms for normal homeostatic plasticity are functional in the Fmr1-KO ex vivo circuits. It is important to note that unlike homeostatic forms of plasticity such as synaptic scaling, regulation of Up-states likely requires retuning of multiple neural loci including at excitatory and inhibitory synapses^78,79,103,104^. Thus the presence of normal homeostatic regulation of Up-states suggests that at least some of the learning rules responsible for regulating network dynamics are functional.

A more stringent test of whether the learning rules that govern network dynamics are operational in FMRP-deficient circuits is whether these circuits undergo normal experience-dependent plasticity. That is, if *Fmr1*^*−/y*^ cortical circuits are capable of adapting to, and learning, the structure of the stimuli they are exposed to. While it is well established that FXS animals exhibit learning deficits, it is often challenging to directly attribute those deficits to local alterations in cortical circuits. A reduced ex vivo approach provides a mean to study microcircuit-level experience-dependent plasticity, while bypassing potential effects associated with sensory hypersensitivity, neuromodulation, attention, and up/down stream alterations. Thus to emulate an experience-dependent learning protocol we asked if cortical circuits can adapt in an experience-dependent manner to the temporal structure of chronically presented stimuli^69^.

In WT slices the network dynamics evoked by a single light pulse differed according to the training history of the slice: the duration of evoked dynamics was longer in slices trained with long intervals compared to slices trained with short intervals (Fig. 6). This finding is consistent with previous acute and organotypic slice experiments that have reported in vitro analogs of learning^65–67,69,105^. In contrast to WT circuits however, *Fmr1*^*−/y*^ slices did not exhibit differential responses between the slices trained with short and long intervals. These results offer the first suggestion that it may be possible to reproduce experience-dependent deficits that characterize FXS in a reduced ex vivo system.

Future studies will have to determine why *Fmr1*^*−/y*^ circuits did not adapt in a stimulus specific manner, and dissect which set of learning rules underlying this deficit. We hypothesize, that this circuit-level experience-dependent deficit may be driven by abnormalities in the ability of multiple learning rules to interact in an orchestrated manner. For example, for computational models of neural networks to converge to stable solutions it is necessary that the multiple learning rules be appropriately tuned in relation to each other. For example in models that contain both homeostatic and associative forms of synaptic plasticity, the learning rates of both learning rules must be appropriately balanced in order to converge to a stable solution^106–108^.

## Conclusion

In agreement with previous studies^24,34,89,90,92,109^, our results further highlight the importance of alterations in local neural dynamics as a major neural phenotype in FXS. A critical issue, however, relates to the underlying cause of the observed changes in neural dynamics. For example, whether the atypical dynamics is the result of “structural” deficits (such as abnormalities in spine number) that persist into adulthood, or rather, of learning rules that govern neuronal properties such as synaptic strength. By studying plasticity of circuit-level dynamics by emulating sensory experience, we established that *Fmr1*^*−/y*^ circuits were robustly able to adapt to increased levels of external drive by decreasing spontaneous Up-states. In contrast to WT circuits, however, *Fmr1*^*−/y*^ circuits did not adapt in an experience-dependent manner to the chronic presentation of structured external inputs. These results suggest that while homeostatic learning rules governing network activity are normal, there may be deficits in associative learning rules or in the ability of homeostatic and associative learning rules to interact in an orchestrated manner. Finally, the ex vivo experience-dependent learning protocol used here provides a potential reduced system to pinpoint the abnormal learning rules in *Fmr1*^*−/y*^ circuits in a targeted manner, while minimizing potential confounds associated with indirect and experiential differences.

## Methods

### Experimental animals

All experiments were conducted in accordance with the US National Institutes of Health guidelines for animal research, and approved by the Chancellor's Animal Research Committee at the University of California, Los Angeles. Wild-type (WT) male mice (*Fmr1*^+*/y*^, Jackson Labs #004828]) and Fmr1-KO female mice (*Fmr1*^*−/−*^, #004624) on the FVB background (FVB.129P2) were used to establish a colony by breeding heterozygous females (*Fmr1*^*−/*+^) and WT males (*Fmr1*^+*/y*^). Mice were housed in the vivarium under a 12-h light/dark cycle.

### Organotypic slice preparation

Organotypic slices were prepared using the interface method^110,111^ from postnatal day 6–7 *Fmr1*^*−/y*^ and *Fmr1*^+*/y*^ littermate male mice. Male animals were used because females in these litters were not *Fmr1* homozygous. Mice were anesthetized with isoflurane and decapitated. As described previously^111^, the brain was removed and placed in chilled cutting media. Coronal slices (400 µm thickness) containing mainly primary auditory cortex were cut using a vibratome and placed on Millipore (Billerica, MA) filters (MillicellCM) with 1 ml of culture media. Culture media was changed 1 and 24 h after cutting, and every 2–3 days thereafter. Cutting media consisted of Eagle’s minimum essential medium (EMEM; catalog number 15-010; MediaTech, Herndon, VA) plus (in mM): 3 MgCl_2_, 10 glucose, 25 HEPES, and 10 Tris base. Culture media consisted of EMEM plus (in mM) 4 glutamine, 0.6 CaCl_2_, 1.85 MgSO_4_, 30 glucose, 30 HEPES, 0.5 ascorbic acid, 20% horse serum, 10 U/I penicillin, and 10 µg/l streptomycin. Slices were incubated in 5% CO_2_ at 35 °C for 7–30 days in vitro (DIV). At the time of brain harvesting a tail sample from each mouse was collected for genotyping (Transnetyx).

### Viral transfection

Viral transfection occurred at 1 or 7 DIV. For two-photon Ca^2+^-imaging, AAV5/9.Syn.GCaMP6f.WPRE.SV40 (UPenn Vector Core, titer: 10^13^ genomes per ml) was delivered at either 1 DIV for the 11–16 DIV developmental study or at 7 DIV for the 25–30 DIV developmental study. For chronic optical stimulation (COS) experiments both AAV9.Syn.GCaMP6f.WPRE.SV40 (UPenn Vector Core, titer: 10^13^ genomes per ml) and AAV9.syn.ChrimsonR-tdtomato.WPRE.BGH (UPenn Vector Core, titer: 10^13^ genomes per ml) were delivered at 7 DIV. Transfection was achieved by gently delivering ~ 1 µl of each viral solution in a glass pipette, with the aid of a manual micromanipulator, to 4–5 different locations in cortical layer (L) II/III. When double transfecting, 1 µl of each virus were mixed and then placed in a single glass pipette for delivery. Experiments were performed two to three weeks after transfection to allow robust viral expression.

### Electrophysiology

All experiments (Ca^2+^-imaging and electrophysiology) were performed at 11–16 or at 25–30 DIV in ACSF composed of (in mM): 125 NaCl, 5.1 KCl, 2.6 MgSO_4_, 26.1 NaHCO_3_, 1 NaH2PO_4_, 25 glucose, and 2.6 CaCl_2_. This ACSF was formulated to match the standard culture media^64^. Whole-cell recordings were made from LII/III regular-spiking, supragranular pyramidal neurons using infrared differential interference contrast visualization at 25–30 DIV. The internal solution for whole-cell recordings contained (in mM) 100 K-gluconate, 20 KCl, 4 ATP-Mg, 10 phospho-creatine, 0.03 GTP-Na, and 10 HEPES and was adjusted to pH 7.3 and 300 mOsm. Temperature was maintained at 32–33 °C, and the ACSF perfusion rate was set to 5–6 ml/min. Only cells that satisfied the following criteria were accepted for analysis: resting membrane potential less than − 60 mV, input resistance between 100 and 300 MΩ, and series resistance of less than 25 MΩ. Cells were discarded if resting membrane potential changed by more than 10 mV during the course of recording.

### Two-photon calcium imaging

Ca^2+^-imaging was performed with a galvo-resonant-scanning two-photon microscope (Neurolabware) controlled by Scanbox acquisition software (https://scanbox.org). A Coherent Chameleon Ultra II Ti:sapphire laser (Cambridge Technologies) was used for GCaMP6f excitation (920 nm). We used a 16 × water-immersion lens (Nikon, 0.8 NA, 3 mm working distance). Image sequences were captured using unidirectional scanning at a frame rate of ~ 15–30 Hz. The size of the recorded imaging field was ~ 520 × 800 µm (512 × 796 pixels). GCaMP6f was used because of its relatively fast kinetics, and the relative changes in somatic fluorescence of GCaMP6f were used as a non-linear readout of the neuronal spiking activity^112^. Regions of interests (ROI) were established in a semi-automated manner, based on manual thresholding of the pairwise pixel correlation (Scanbox). ∆F/F was calculated as (F(t) − F0))/F0, where F(t) was the raw fluorescence filtered with a median filter with a window of 1 s. F0 was the running median F(t) over the previous 10 s window.

### Up-state identification and analysis of spatiotemporal structure of neural dynamics from Ca^2+^ imaging data

For each recorded slice, potential Up-states were identified based on a threshold set at 1.5 above the mean z-scored ΔF/F trace of all neurons. If these events remained above threshold for at least one second, they were classified as Up-states. Onsets and offsets of the population based Up-states were marked, and the ΔF/F profiles of each neuron between one second before Up-state onset and one second after Up-state offset were extracted (Fig. Supplement 2A). The 1 s baseline window is necessary in order for the correlation to pick up deviations from rest. A single Up-state was represented in an NxT matrix, where N corresponds to the number of cells and T as the number of frames. To quantify the overall similarity of these trajectories, we calculated the mean of the pairwise 2D correlation between all Up-states within a slice. We excluded experiments that had less than four Up-states (mean 9 ± 0.97) within the 5-min window of Ca^2+^ imaging. Importantly, and because the T values of the NxT matrices can be different (differences in Up-state duration), we used the window corresponding to the duration of the longest Up-state in a given pair—in other words the overall duration of Up-states contributed to the measure of Up-state similarity. In order to do so, the shorter Up-state was padded with zeros. This similarity index was contrasted with two types of shuffled controls: (1) within-shuffle, in which the neurons within a given Up-state were shuffled; and (2) between-shuffle, in which the same cell maintained its position but was shuffled between Up-states (Fig. 3A). Additionally, we performed clustering analysis on the mean ΔF/F over time of each cell in an Up-state. For this, the NxT matrix was collapsed across time, and each Up-state was represented by a vector of size N. We also performed principal component analysis (PCA) on the concatenated Up-states, and plotted the scores of the top three principal components. This analysis, however, was only used for visualization purposes (Fig. Supplement 2B,C).

To extract the time (latencies) of the Ca^2+^ trace peaks we used the *findpeaks* MATLAB function, and used the median of these peak times across all segmented cells within a slice and all ten light evoked test trials.

### Optical stimulation

For the all-optical homeostatic plasticity experiments, ex vivo slices that co-expressed ChrimsonR and GCaMP6f were used. To reduce variability, control and experimental groups relied on "sister” slices, derived from the same batch of animals (littermates), maintained with the same culture medium and serum, placed in the same incubator and virally transfected in the same session. In each experiment, two slices (from the same animal) of each genotype (*Fmr1*^+*/y*^ or *Fmr1*^*−/y*^) were placed in the “stimulating incubator” at 21–25 DIV (four slices from two littermate mice). One slice per genotype received chronic optical stimulation (COS) via a red LED (Super Bright LEDs, 630 nm wavelength) while the other was kept in the same incubator but did not receive COS. Optical stimulation consisted of a 50 ms flash of red light driven by two 1.5 V batteries and yielded an output of ~ 0.1 mW/mm^2^ delivered every 30 s for 3–4 days. Immediately following COS slices were transferred on their intact filters to a custom-built chamber on the stage of the 2P rig and Ca^2+^ imaging was performed at 25–30 DIV (Fig. 5A,B). For acute optical stimulation during Ca^2+^ imaging experiments, we used an external LED (Thorlabs 625 nm, M625L4) with a longpass filter (Thorlabs 600 nm, FEL0600) to deliver full-field optogenetic manipulation while minimizing light leakage into the green photomultiplier tube (Fig. Supplement 3). For Interval learning experiments, a similar stimulation protocol was used, however, the light delivery consisted of two pulses of 50 ms separated by 250 ms (short group) or by either 750 or 1,000 ms (long group) every 60 s for 24 h (Fig. 6A).

### Figures

All figures plotted using box-and-whisker plots, box represents 25–75% (interquartile) percentile range, whiskers represent the range of the non-outlier data points. Outliers (squares) are those that fall 1.5 times the interquartile range above or below the box edges. The line within the box represents the median. ImageJ was used to create immunohistochemistry figures.

### Statistics

All statistical analyses were done using MATLAB. Statistical tests for normality (Lilliefors test) were performed on each data set, and depending on whether the data significantly deviated from normality (p < 0.05) or did not deviate from normality (p > 0.05) appropriate non-parametric or parametric tests were performed. The statistical tests performed are mentioned in the text and the legends. Parametric analyses relied on students t-test and one- or two-way ANOVAs. The Mann–Whitney (RankSum function in MATLAB) was used for nonparametric comparisons. For the data in Figs. 3 and 4 the similarity indices were Fisher transformed for presentation and statistical tests.

## Supplementary information

Supplementary Figures.

## Data Availability

The custom-written MATLAB scripts and data are available from the corresponding authors upon reasonable request.

## References

[1] Wassink TH, Piven J, Patil SR (2001). Chromosomal abnormalities in a clinic sample of individuals with autistic disorder. Psychiatr. Genet..

[2] Reddy KS (2005). Cytogenetic abnormalities and fragile-X syndrome in autism spectrum disorder. BMC Med. Genet..

[3] Darnell JC, Klann E (2013). The translation of translational control by FMRP: Therapeutic targets for FXS. Nat. Neurosci..

[4] Contractor A, Klyachko VA, Portera-Cailliau C (2015). Altered neuronal and circuit excitability in Fragile X syndrome. Neuron.

[5] Consortium, D.-B. F. X (1994). Fmr1 knockout mice: A model to study fragile X mental retardation. The Dutch–Belgian Fragile X consortium. Cell.

[6] Hinton VJ, Brown WT, Wisniewski K, Rudelli RD (1991). Analysis of neocortex in three males with the fragile X syndrome. Am. J. Med. Genet..

[7] Irwin SA, Galvez R, Greenough WT (2000). Dendritic spine structural anomalies in fragile-X mental retardation syndrome. Cereb. Cortex..

[8] Nimchinsky EA, Oberlander AM, Svoboda K (2001). Abnormal development of dendritic spines in FMR1 knock-out mice. J. Neurosci..

[9] Cruz-Martin A, Crespo M, Portera-Cailliau C (2010). Delayed stabilization of dendritic spines in fragile X mice. J. Neurosci..

[10] Portera-Cailliau C (2012). Which comes first in fragile X syndrome, dendritic spine dysgenesis or defects in circuit plasticity?. Neuroscientist.

[11] Galvez R, Gopal AR, Greenough WT (2003). Somatosensory cortical barrel dendritic abnormalities in a mouse model of the fragile X mental retardation syndrome. Brain Res..

[12] Patel AB, Loerwald KW, Huber KM, Gibson JR (2014). Postsynaptic FMRP promotes the pruning of cell-to-cell connections among pyramidal neurons in the L5A neocortical network. J. Neurosci..

[13] Bear MF, Huber KM, Warren ST (2004). The mGluR theory of fragile X mental retardation. Trends Neurosci..

[14] Kim H, Gibboni R, Kirkhart C, Bao S (2013). Impaired critical period plasticity in primary auditory cortex of fragile X model mice. J. Neurosci..

[15] Soden ME, Chen L (2010). Fragile X protein FMRP is required for homeostatic plasticity and regulation of synaptic strength by retinoic acid. J. Neurosci..

[16] Deng PY (2013). FMRP regulates neurotransmitter release and synaptic information transmission by modulating action potential duration via BK channels. Neuron.

[17] Deng PY, Sojka D, Klyachko VA (2011). Abnormal presynaptic short-term plasticity and information processing in a mouse model of fragile X syndrome. J. Neurosci..

[18] Hu H (2008). Ras signaling mechanisms underlying impaired GluR1-dependent plasticity associated with fragile X syndrome. J. Neurosci..

[19] Antar LN, Li C, Zhang H, Carroll RC, Bassell GJ (2006). Local functions for FMRP in axon growth cone motility and activity-dependent regulation of filopodia and spine synapses. Mol. Cell Neurosci..

[20] Tian Y (2017). Loss of FMRP impaired hippocampal long-term plasticity and spatial learning in rats. Front. Mol. Neurosci..

[21] Bureau I, Shepherd GM, Svoboda K (2008). Circuit and plasticity defects in the developing somatosensory cortex of FMR1 knock-out mice. J. Neurosci..

[22] Meredith RM, Holmgren CD, Weidum M, Burnashev N, Mansvelder HD (2007). Increased threshold for spike-timing-dependent plasticity is caused by unreliable calcium signaling in mice lacking fragile X gene FMR1. Neuron.

[23] Hanson JE, Madison DV (2007). Presynaptic FMR1 genotype influences the degree of synaptic connectivity in a mosaic mouse model of fragile X syndrome. J. Neurosci..

[24] Gibson JR, Bartley AF, Hays SA, Huber KM (2008). Imbalance of neocortical excitation and inhibition and altered UP states reflect network hyperexcitability in the mouse model of fragile X syndrome. J. Neurophysiol..

[25] Brown MR (2010). Fragile X mental retardation protein controls gating of the sodium-activated potassium channel Slack. Nat. Neurosci..

[26] Gross C, Yao X, Pong DL, Jeromin A, Bassell GJ (2011). Fragile X mental retardation protein regulates protein expression and mRNA translation of the potassium channel Kv4.2. J. Neurosci..

[27] Brager DH, Akhavan AR, Johnston D (2012). Impaired dendritic expression and plasticity of h-channels in the fmr1(−/y) mouse model of fragile X syndrome. Cell Rep..

[28] Lee HY, Jan LY (2012). Fragile X syndrome: mechanistic insights and therapeutic avenues regarding the role of potassium channels. Curr. Opin. Neurobiol..

[29] Routh BN, Johnston D, Brager DH (2013). Loss of functional A-type potassium channels in the dendrites of CA1 pyramidal neurons from a mouse model of fragile X syndrome. J. Neurosci..

[30] Zhang Y (2014). Dendritic channelopathies contribute to neocortical and sensory hyperexcitability in Fmr1(−/y) mice. Nat. Neurosci..

[31] Ferron L, Nieto-Rostro M, Cassidy JS, Dolphin AC (2014). Fragile X mental retardation protein controls synaptic vesicle exocytosis by modulating N-type calcium channel density. Nat. Commun..

[32] Harlow EG (2010). Critical period plasticity is disrupted in the barrel cortex of FMR1 knockout mice. Neuron.

[33] Paluszkiewicz SM, Martin BS, Huntsman MM (2011). Fragile X syndrome: The GABAergic system and circuit dysfunction. Dev. Neurosci..

[34] Goncalves JT, Anstey JE, Golshani P, Portera-Cailliau C (2013). Circuit level defects in the developing neocortex of Fragile X mice. Nat. Neurosci..

[35] Curia G, Papouin T, Seguela P, Avoli M (2009). Downregulation of tonic GABAergic inhibition in a mouse model of fragile X syndrome. Cereb. Cortex..

[36] He Q, Nomura T, Xu J, Contractor A (2014). The developmental switch in GABA polarity is delayed in fragile X mice. J. Neurosci..

[37] Vislay RL (2013). Homeostatic responses fail to correct defective amygdala inhibitory circuit maturation in fragile X syndrome. J. Neurosci..

[38] Goel A (2018). Impaired perceptual learning in a mouse model of Fragile X syndrome is mediated by parvalbumin neuron dysfunction and is reversible. Nat. Neurosci..

[39] D'Hulst C (2009). Expression of the GABAergic system in animal models for fragile X syndrome and fragile X associated tremor/ataxia syndrome (FXTAS). Brain Res..

[40] Zhang N (2017). Decreased surface expression of the delta subunit of the GABAA receptor contributes to reduced tonic inhibition in dentate granule cells in a mouse model of fragile X syndrome. Exp. Neurol..

[41] Kang JY (2017). Deficits in the activity of presynaptic gamma-aminobutyric acid type B receptors contribute to altered neuronal excitability in fragile X syndrome. J. Biol. Chem..

[42] Telias M, Segal M, Ben-Yosef D (2016). Immature responses to GABA in Fragile X neurons derived from human embryonic stem cells. Front. Cell Neurosci..

[43] He Q (2019). Critical period inhibition of NKCC1 rectifies synapse plasticity in the somatosensory cortex and restores adult tactile response maps in fragile X mice. Mol. Psychiatry.

[44] Antoine MW, Langberg T, Schnepel P, Feldman DE (2019). Increased excitation-inhibition ratio stabilizes synapse and circuit excitability in four autism mouse models. Neuron.

[45] Nelson SB, Valakh V (2015). Excitatory/inhibitory balance and circuit homeostasis in autism spectrum disorders. Neuron.

[46] Belmonte MK, Bourgeron T (2006). Fragile X syndrome and autism at the intersection of genetic and neural networks. Nat. Neurosci..

[47] Buonomano DV, Merzenich MM (1998). Cortical plasticity: From synapses to maps. Annu. Rev. Neurosci..

[48] Feldman DE, Brecht M (2005). Map plasticity in somatosensory cortex. Science.

[49] Meaney MJ (2001). Maternal care, gene expression, and the transmission of individual differences in stress reactivity across generations. Annu. Rev. Neurosci..

[50] Rioult-Pedotti MS, Friedman D, Donoghue JP (2000). Learning-induced LTP in neocortex. Science.

[51] Wang L, Fontanini A, Maffei A (2012). Experience-dependent switch in sign and mechanisms for plasticity in layer 4 of primary visual cortex. J. Neurosci..

[52] Kirkwood A, Lee H-K, Bear MF (1995). Co-regulation of long-term potentiation and experience-dependent synaptic plasticity in visual cortex by age and experience. Nature.

[53] Arroyo ED, Fiole D, Mantri SS, Huang C, Portera-Cailliau C (2019). Dendritic spines in early postnatal Fragile X mice are insensitive to novel sensory experience. J. Neurosci..

[54] Oddi D (2014). Early social enrichment rescues adult behavioral and brain abnormalities in a mouse model of fragile X syndrome. Neuropsychopharmacology.

[55] Roy S, Watkins N, Heck D (2012). Comprehensive analysis of ultrasonic vocalizations in a mouse model of Fragile X syndrome reveals limited, call type specific deficits. PLoS ONE.

[56] Zupan B, Toth M (2008). Wild-type male offspring of fmr-1+/− mothers exhibit characteristics of the Fragile X Phenotype. Neuropsychopharmacology.

[57] Spencer CM, Alekseyenko O, Serysheva E, Yuva-Paylor LA, Paylor R (2005). Altered anxiety-related and social behaviors in the Fmr1 knockout mouse model of fragile X syndrome. Genes Brain Behav..

[58] Zupan B, Sharma A, Frazier A, Klein S, Toth M (2016). Programming social behavior by the maternal fragile X protein. Genes Brain Behav..

[59] Restivo L (2005). Enriched environment promotes behavioral and morphological recovery in a mouse model for the fragile X syndrome. Proc. Natl. Acad. Sci. USA.

[60] Bolz J (1994). Cortical circuitry in a dish. Curr. Opin. Neurobiol..

[61] Echevarria D, Albus K (2000). Activity-dependent development of spontaneous bioelectric activity in organotypic cultures of rat occipital cortex. Brain Res. Dev. Brain Res..

[62] De Simoni A, Griesinger CB, Edwards FA (2003). Development of rat CA1 neurones in acute versus organotypic slices: Role of experience in synaptic morphology and activity. J. Physiol..

[63] Johnson HA, Buonomano DV (2007). Development and plasticity of spontaneous activity and up states in cortical organotypic slices. J. Neurosci..

[64] Goel A, Buonomano DV (2013). Chronic electrical stimulation homeostatically decreases spontaneous activity, but paradoxically increases evoked network activity. J. Neurophysiol..

[65] Dranias MR, Westover MB, Cash SS, VanDongen AMJ (2015). Stimulus information stored in lasting active and hidden network states is destroyed by network bursts. Front. Integr. Neurosci..

[66] Chubykin AA, Roach EB, Bear MF, Shuler MGH (2013). A cholinergic mechanism for reward timing within primary visual cortex. Neuron.

[67] Hyde RA, Strowbridge BW (2012). Mnemonic representations of transient stimuli and temporal sequences in the rodent hippocampus in vitro. Nat. Neurosci..

[68] Isomura T, Kotani K, Jimbo Y (2015). Cultured cortical neurons can perform blind source separation according to the free-energy principle. PLoS Comput. Biol..

[69] Goel A, Buonomano DV (2016). Temporal interval learning in cortical cultures is encoded in intrinsic network dynamics. Neuron.

[70] Steriade M, McCormick D, Sejnowski T (1993). Thalamocortical oscillations in the sleeping and aroused brain. Science.

[71] Timofeev I, Grenier F, Bazhenov M, Sejnowski TJ, Steriade M (2000). Origin of slow cortical oscillations in deafferented cortical slabs. Cereb. Cortex..

[72] MacLean JN, Watson BO, Aaron GB, Yuste R (2005). Internal dynamics determine the cortical response to thalamic stimulation. Neuron.

[73] Sadovsky AJ, MacLean JN (2014). Mouse visual neocortex supports multiple stereotyped patterns of microcircuit activity. J. Neurosci..

[74] Sanchez-Vives MV, McCormick DA (2000). Cellular and network mechanisms of rhythmic recurrent activity in neocortex. Nat. Neurosci..

[75] Neske GT, Patrick SL, Connors BW (2015). Contributions of diverse excitatory and inhibitory neurons to recurrent network activity in cerebral cortex. J. Neurosci..

[76] Bartram J (2017). Cortical Up states induce the selective weakening of subthreshold synaptic inputs. Nat. Commun..

[77] Luczak A, Bartho P, Marguet SL, Buzsaki G, Harris KD (2007). Sequential structure of neocortical spontaneous activity in vivo. Proc. Natl. Acad. Sci. USA.

[78] Jercog D (2017). UP-DOWN cortical dynamics reflect state transitions in a bistable network. eLife.

[79] Shu Y, Hasenstaub A, McCormick DA (2003). Turning on and off recurrent balanced cortical activity. Nature.

[80] Motanis H, Buonomano DV (2015). Delayed in vitro development of up states but normal network plasticity in Fragile X Circuits. Eur. J. Neurosci..

[81] Kroener S, Chandler LJ, Phillips PE, Seamans JK (2009). Dopamine modulates persistent synaptic activity and enhances the signal-to-noise ratio in the prefrontal cortex. PLoS ONE.

[82] Tononi G, Cirelli C (2003). Sleep and synaptic homeostasis: A hypothesis. Brain Res. Bull..

[83] Diekelmann S, Born J (2010). The memory function of sleep. Nat. Rev. Neurosci..

[84] Marshall L, Helgadóttir H, Mölle M, Born J (2006). Boosting slow oscillations during sleep potentiates memory. Nature.

[85] Vyazovskiy VV, Cirelli C, Pfister-Genskow M, Faraguna U, Tononi G (2008). Molecular and electrophysiological evidence for net synaptic potentiation in wake and depression in sleep. Nat. Neurosci..

[86] Sirota A, Buzsáki G (2007). Interaction between neocortical and hippocampal networks via slow oscillations. Thalamus Relat. Syst..

[87] Destexhe A, Hughes SW, Rudolph M, Crunelli V (2007). Are corticothalamic ‘up’ states fragments of wakefulness?. Trends Neurosci..

[88] Westmark CJ (2016). APP causes hyperexcitability in Fragile X Mice. Front. Mol. Neurosci..

[89] Hays SA, Huber KM, Gibson JR (2011). Altered neocortical rhythmic activity states in Fmr1 KO mice are due to enhanced mGluR5 signaling and involve changes in excitatory circuitry. J. Neurosci..

[90] Berzhanskaya J (2017). Disrupted cortical state regulation in a rat model of Fragile X Syndrome. Cereb. Cortex..

[91] He CX (2017). Tactile defensiveness and impaired adaptation of neuronal activity in the fmr1 knock-out mouse model of autism. J. Neurosci..

[92] Berzhanskaya J, Phillips MA, Shen J, Colonnese MT (2016). Sensory hypo-excitability in a rat model of fetal development in Fragile X Syndrome. Sci. Rep..

[93] Bazhenov M, Timofeev I, Steriade M, Sejnowski TJ (2002). Model of thalamocortical slow-wave sleep oscillations and transitions to activated States. J. Neurosci..

[94] Johnson HA, Buonomano DV (2009). A method for chronic stimulation of cortical organotypic cultures using implanted electrodes. J. Neurosci. Methods.

[95] Corner MA, van Pelt J, Wolters PS, Baker RE, Nuytinck RH (2002). Physiological effects of sustained blockade of excitatory synaptic transmission on spontaneously active developing neuronal networks—An inquiry into the reciprocal linkage between intrinsic biorhythms and neuroplasticity in early ontogeny. Neurosci. Biobehav. Rev..

[96] Martin BS, Huntsman MM (2012). Pathological plasticity in fragile X syndrome. Neural. Plast..

[97] Telias M (2019). Molecular mechanisms of synaptic dysregulation in Fragile X Syndrome and autism spectrum disorders. Front. Mol. Neurosci..

[98] Till SM (2012). Altered maturation of the primary somatosensory cortex in a mouse model of fragile X syndrome. Hum. Mol. Genet..

[99] Testa-Silva G (2012). Hyperconnectivity and slow synapses during early development of medial prefrontal cortex in a mouse model for mental retardation and autism. Cereb. Cortex..

[100] Pilpel Y (2009). Synaptic ionotropic glutamate receptors and plasticity are developmentally altered in the CA1 field of Fmr1 knockout mice. J. Physiol..

[101] Toft AK, Lundbye CJ, Banke TG (2016). Dysregulated NMDA-receptor signaling inhibits long-term depression in a mouse model of Fragile X Syndrome. J. Neurosci..

[102] Lieb-Lundell CC (2016). Three faces of Fragile X. Phys. Ther..

[103] Haider B, Duque A, Hasenstaub AR, McCormick DA (2006). Neocortical network activity in vivo is generated through a dynamic balance of excitation and inhibition. J. Neurosci..

[104] Brunel N (2000). Dynamics of networks of randomly connected excitatory and inhibitory spiking neurons. J. Physiol. Paris.

[105] Johnson HA, Goel A, Buonomano DV (2010). Neural dynamics of in vitro cortical networks reflects experienced temporal patterns. Nat. Neurosci..

[106] Liu JK, Buonomano DV (2009). Embedding multiple trajectories in simulated recurrent neural networks in a self-organizing manner. J. Neurosci..

[107] Fiete IR, Senn W, Wang CZH, Hahnloser RHR (2010). Spike-time-dependent plasticity and heterosynaptic competition organize networks to produce long scale-free sequences of neural activity. Neuron.

[108] Lazar A, Pipa G, Triesch J (2009). SORN: A self-organizing recurrent neural network. Front. Comput. Neurosci..

[109] Ronesi JA (2012). Disrupted Homer scaffolds mediate abnormal mGluR5 function in a mouse model of fragile X syndrome. Nat. Neurosci..

[110] Stoppini L, Buchs PA, Muller D (1991). A simple method for organotypic cultures of nervous tissue. J. Neurosci. Methods.

[111] Buonomano DV (2003). Timing of neural responses in cortical organotypic slices. Proc. Natl. Acad. Sci. USA.

[112] Chen TW (2013). Ultrasensitive fluorescent proteins for imaging neuronal activity. Nature.

