# Supplementary material for: Decreased reproducibility and abnormal experience-dependent plasticity of network dynamics in Fragile X circuits
Source: Sci Rep. 2020 Sep 3;10:14535. doi: 10.1038/s41598-020-71333-y (PMC7471942; doi:10.1038/s41598-020-71333-y)
Supplement: Supplementary file 1 — Supplementary Figures. [file 41598_2020_71333_MOESM1_ESM.docx]

**Decreased reproducibility and abnormal**

**experience-dependent plasticity of network**

**dynamics in Fragile X circuits**

**Helen Motanis and Dean Buonomano***

**Affiliation:** Departments of Neurobiology and Psychology, and Integrative Center for Learning and Memory, University of California, Los Angeles, CA 90095, USA

**Corresponding author:**

**Dean Buonomano**

**Address:**  Departments of Neurobiology and Psychology, and Integrative Center for Learning and Memory, University of California, 630 Charles E Young Dr S, Center for Health Sciences Building, Los Angeles, CA 90095, USA

**e-mail:** [dbuono@ucla.edu](mailto:dbuono@ucla.edu)

**Supplementary Figure 1**


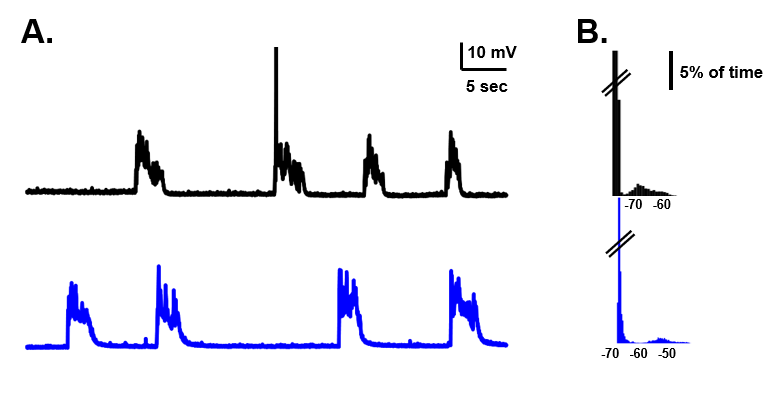


**Supplementary 1: Up-states are healthy network-wide events**

**A.** Whole-cell recordings of Up-states in neurons from two WT *ex vivo* slices at 25 DIV. **B.** Histogram of the voltage of traces shown in A demonstrating the characteristic bimodal distribution of voltage.

**Supplementary Figure 2**


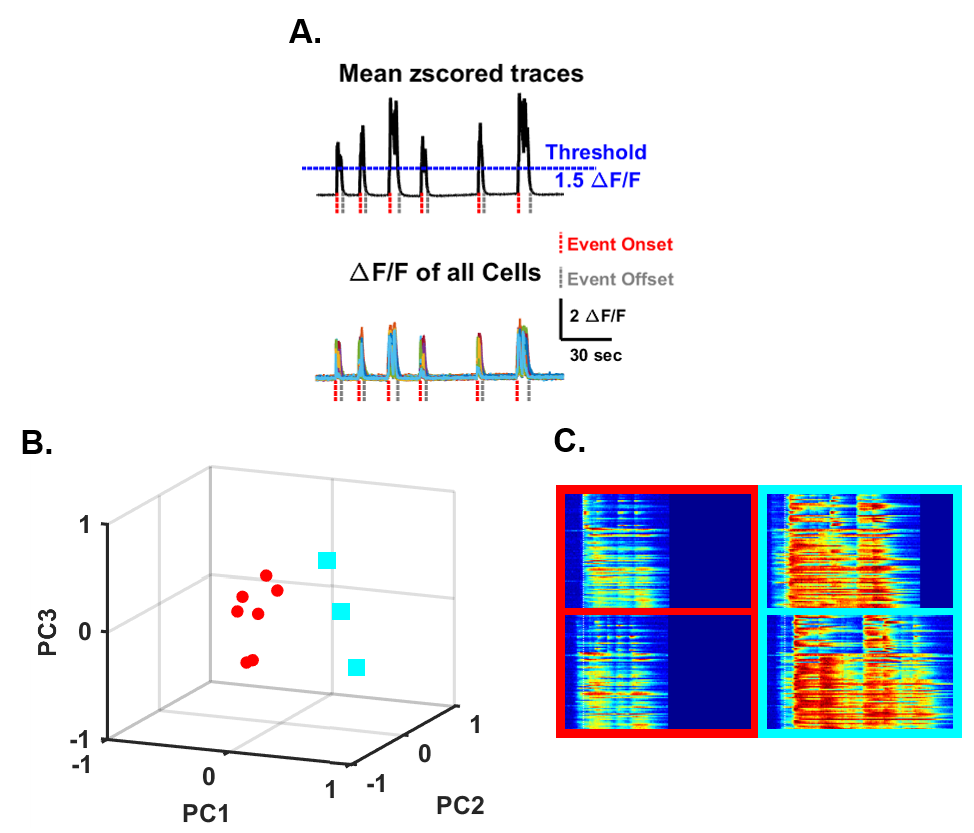


**Supplementary 2: WT circuits exhibit distinct patterns of activity**

**A.** Example of the mean z-scored trace of all neurons from a *Fmr1^+/y^* circuit (top). Threshold used to detect Up-states was 1.5. Bottom trace shows GCaMP6f ΔF/F of all neurons in the above *Fmr1^+/y^* circuit. Onsets of Up-states are marked with dashed red lines (first lines) while offsets are marked with dashed gray lines (second lines). **B.** First three principal components from an *Fmr1^+/y^* circuit shows two distinct populations (red circles vs. cyan squares). **C.** Four Up-states from the example *Fmr1^+/y^* slice in B. The two Up-states on the left belong to the same cluster (red circles) while the two Up-states on the right belong to a different cluster (cyan squares).

**Supplementary Figure 3**


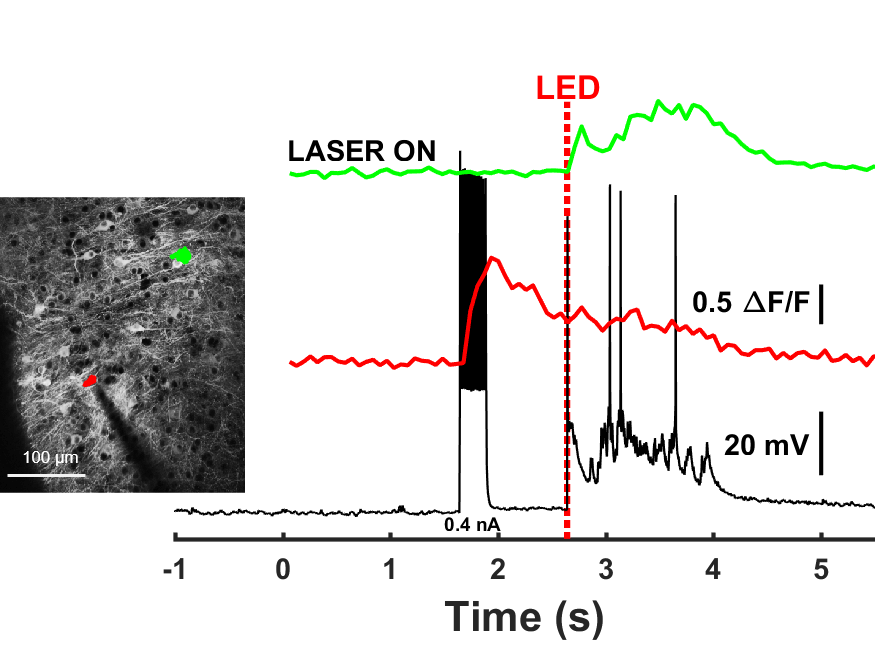


**Supplementary 3: Two-photon imaging does not activate Chrimson-positive neurons.**

The top two traces (green, upper top trace) and red (middle trace) are the ΔF/F profiles of two neurons, red (left) and green (right) ROIs in inset. The black trace (bottom) is the whole-cell recording corresponding to the red ΔF/F trace (middle trace) (whole-cell recording electrode shadow is visible in the inset). At laser onset there was no clear change in membrane potential (black trace – bottom trace). In response to a 0.4 nA depolarizing step the cell produced multiple spikes (black trace – bottom trace) and elicited a sharp ΔF/F signal in only the corresponding ΔF/F profile (red trace – middle trace). Note that the green trace (top trace) does not have any ΔF/F change as a result of the 0.4 nA depolarizing step in a nearby red cell (left cell). In response to a 50 ms red light pulse (625 nm) both cells were activated, and an evoked Up-state was elicited (top two traces). Note that the partially saturated Ca^2+-^signal only partially detects single spikes in the red cell.
